# Assessment of a Score’s Performance in Predicting Positive Culture Studies in Preterm Neonates with Clinical Suspicion of Sepsis

**DOI:** 10.34763/jmotherandchild.20212502.d-21-00019

**Published:** 2022-04-01

**Authors:** Ricardo Barreto Mota, Paulo Soares, Hercília Guimarães

**Affiliations:** 1 Neonatal Intensive Care Unit, Centro Hospitalar e Universitário São João, Porto, Portugal; 2 Faculty of Medicine of University of Porto, Porto Portugal

**Keywords:** Sepsis, preterm, organ failure, platelets, c-reactive protein

## Abstract

The goal of this study is to assess the use of a score composed of markers of inflammation and organ failure to predict positive cultures for preterm newborns with clinical suspicion of late-onset sepsis. The score was calculated at the first suspicion and 24–48 hours later. We retrospectively compared score results between neonates with positive and negative cultures. Neonates with positive cultures had a significantly higher score at the second instance; the receiver operator characteristics curve presented an area under the curve of 0.798 (*p*=0.007). A score for early prediction of sepsis could be an important tool for prognostic improvement in the future.

## Introduction

Late-onset sepsis (LOS) is a significant cause of death in the neonatal intensive care unit (NICU).[1] Its incidence is inversely correlated with gestational age and birthweight, which may be explained by immaturity, long ventilation courses and invasive procedures, amongst several other factors. [2, 3] In preterm infants, the reported mortality is between 18% and 36%.[4] There is no international consensus on the definition of sepsis in newborns, potentially leading to overdiagnosis and overtreatment with antibiotics.[5] In preterm infants, this problem is enhanced due to nonspecific presentations, such as acute alterations in the respiratory pattern, bradycardia, lethargy or feeding intolerance.[6] The definitive diagnosis of sepsis is traditionally established by the presence of positive blood, urine, or cerebrospinal fluid (CSF) cultures. The same difficulty arises when defining prognosis factors through organ failure scores, since the validated scores for adult and pediatric populations do not seem to be accurate for neonates.[5, 6] Wynn and Polin described a score accessing organ dysfunction in preterm neonates with LOS, reporting it as being predictive of mortality with a similar performance to the Sequential Organ Failure Assessment score in adults.[7] This was an important first step in classifying at-risk neonates with objective measures, despite, as of this timing, only having been applied in preterm neonates with LOS to assess mortality risk. With our study, we aim to assess the performance of a score, based on inflammation and organ failure markers, to predict positivity of cultures in preterm newborns with clinical suspicion of LOS.

## Material and methods

We conducted a retrospective observational study in which we included all neonates born with a gestational age less than 32 weeks with a clinical suspicion of sepsis between 72 hours and 30 days of age that led to the decision to initiate empirical antibiotic therapy. Both inborn and outborn neonates admitted during the three-year period between 1 January 2017 and 31 December 2019 were included. Only the first episode of suspected sepsis was considered. Clinical and analytical data registered at the timing of suspicion, and 24 to 48 hours later, were collected. The analysed parameters were serum white blood cell (WBC) count, platelet (PLT) count, C-reactive protein (CRP), ventilation need, and SpO2/FiO2 ratio (S/F). We attributed a score to each parameter and summed the score of all parameters to obtain a final score. The parameters were scored according to the following criteria:

- WBC:

o score of 0 if between 4x10^9/L and 20x10^9/L,
o score of 1 if lower than 4x10^9/L or higher than 20x10^9/L/;

- PLT:

o score of 0 if above 150x10^9/L,
o score of 1 if 100-150x10^9/L,
o score of 2 if 50-100x10^9/L,
o score of 3 if below 50,000x10^9/L;

- CRP:

o score of 0 if below 10mg/L,
o score of 1 if 10-30mg/L,
o score of 2 if 30-50mg/L,
o score of 3 if above 50mg/L;

- Ventilation support:

o score of 0 if no ventilator support needed;
o score of 1 if on non-invasive ventilation;
o score of 2 if on invasive mechanical ventilation;

- S/F:

o score of 0 if above 300,
o score of 1 if 200–300,
o score of 2 of 150–200,
o score of 3 if 100–150,
o score of 4 if below 100.

The cut-offs for WBC and PLT and the 10mg/L CRP cut-offs were based on national neonatal sepsis consensus and on other studies on neonatal sepsis.[8, 9] The remaining CRP cut-offs were based on an empirical severity classification employed at our unit. We then compared these parameters and scores between neonates who had a positive culture study (PCS) and those who did not. As culture studies, we included blood, cerebrospinal, and urine cultures collected at the timing of suspicion. Statistical data was conducted using IBM SPSS version 26. The study was approved by our institution’s ethics committee.

## Results

Comparison between neonates with and without PCS are reported in table 1. The most common microorganism was *Staphylococcus epidermidis* (12, 66.6%). No deaths were reported. Platelet count was significantly lower in patients with PCS both at suspicion (420 versus 230x10^9/L, *p*=0.024) and at the second timing (395 versus 153x10^9/L, *p*=0.020). C-reactive protein (CRP) was significantly higher in the second timing (5.8 versus 40.8 mg/L, *p*=0.009), but not at suspicion. Neonates with PCS were more frequently intubated between the first and second timing (*p*=0.006). No statistically significant difference was observed when comparing scores measured at suspicion, but when measured 24 to 48 hours later, neonates with PCS presented a significantly higher score (5 vs 2; *p*=0.021). The Receiver Operator Characteristics Curve (ROCC) for score at the second timing showed an area under the curve (AUC) of 0.798 (95% confidence interval 0.63-0.97; *p*=0.007). Regarding platelet count, the AUC was of 0.892 (95% CI 0.78-1, *p*<0.001) at the second timing and of 0.767 (95% CI 0.60-0.94, *p*=0.011) at suspicion. CPR value at the second timing had an AUC of 0.807 (95% CI 0.64-0.98, p=0.005). When comparing respiratory and analytical scores separately, only analytical score calculated at the second timing was significantly different, being higher in neonates with PCS (3 vs 1, *p*<0.001), with a calculated AUC of 0.800 (95% CI 0.62-0.98, *p*=0.006).

## Discussion

Our score reported an AUC of 0.798 when measured 24 to 48 hours after the initial clinical suspicion, making it a possible clinical tool to predict microbiologically confirmed sepsis. However, the strength of this score appears to rely mainly on its analytical parameters. CRP was higher in the second timing in neonates with PCS, but not at suspicion. This is coherent with the fact that CRP levels reach their peak 10 to 12 hours after onset of infection.[10] Other studies report similar results regarding ROC curves AUC when assessing CRP value 24 hours after suspicion[9], supporting the hypothesis that CRP has a better sensitivity for LOS at 24 hours. Platelet count was the only marker that, judging by its AUC curve, appeared to have early predictive value. Beltempo and colleagues analysed the predictability of platelet levels below 100x10^9/L at 24 hours, describing a low sensitivity of 12% for LOS prediction [9]. Hornik and colleagues analysed the total platelet count at timing of suspicion, describing an AUC of 0.606.[8] We assessed total platelet count at suspicion and at 24 hours, finding a higher AUC than that reported in these two studies. Our study has, however, a much smaller sample size. This is a small retrospective study, with its inherent limitations. Some of the data used to calculate the score, including SpO2/FiO2 ratio and mechanical ventilation, were collected from physical records with variable reliability regarding timing relative to clinical suspicion. Due to this study’s retrospective nature, we could not access clinical signs based on past records in an objective, normalised manner possible for statistical analysis without significant bias, especially in a small sample size. A future prospective study would benefit from the inclusion of these data. While we believe the inclusion of aminergic support would be of value in such study, it was not included since, according to our records, only one neonate was submitted to this treatment in the analysed timings. The small sample limited us from comparing between types of agents, source of biological sample, and timing between collection and microbiological positivity. A larger, long-term prospective study, with timely, accurate measures of clinical and analytical parameters, is needed. It would also be an opportunity to analyse timing between collection and microbiological positivity and to study the applicability of a score in different types of infections.

## Conclusion

Platelet count appears to have early predictive value for PCS in LOS. Our score’s strength relies mainly on analytical inflammatory markers. Despite our study’s limitations, we consider it to be a steppingstone to future scores and diagnostic tools in LOS in preterm neonates. A reliable score for early prediction of sepsis could be an important tool for prognostic improvement in the future.

## Figures and Tables

**Table 1:** Score comparison between groups and Receiver Operator Characteristics (ROC) Curve results for significant parameters. *Mann-Whitney U test; †χ2 test; ‡Fisher exact test; GA – gestational age, WBC – white blood count, PLT – platelet, CRP – C-reactive protein, AUC – area under the curve

Parameters	Cultural Study (CS)			ROC curve	
	Negative (n=15)	Positive (n=18)	*p* value	AUC (95% CI)	*p* value
GA, median weeks, [inter-quartile range (IQR)] Birthweight, median grams [IQR]	27.6 [26.6-29.4] 1030 [855-1400]	28.2 [27.6-30.3] 1043 [940-1360]	-	-	-
Median 1^st^ Minute Apgar Score	6	7			
Median 5^th^ Minute Apgar Score	7	8			
Median 10^th^ Minute Apgar Score	8	8			
Post Menstrual Age at Sepsis Suspicion, Median weeks [IQR]	29.3 [28.1-31.6]	30.6 [29.4-32.1]			
WBC count at suspicion, median x 10^9/L [IQR]	21.3 [10.5-23.9]	16.3 [9-25.1]	0.391*	-	-
PLT count at suspicion, median x 10^9/L [IQR]	420 [301-549]	230 [170-325]	0.024*	0.767 (0.60-0.94)	0.011
CRP at suspicion, median mg/L [IQR]	9.5 [2.2-18.4]	17.5 [4.9-39.9]	0.215*	-	-
WBC at 2nd timing, median x 10^9/L [IQR]	17.1 [8.8-23-3]	14 [10.2-20.3]	0.862*	-	-
PTL at 2nd timing, median x 10^9/L [IQR]	395 [222-529]	153 [99-288]	0.02*	0.892 (0.78-1)	<0.001
CPR at 2nd timing, median mg/L [IQR]	5.8 [1.1-14.5]	40.8 [14.9-82.4]	0.009*	0.807 (0.64-0.98)	0.005
Score at suspicion, median	2.00	3.00	1.000*	-	-
Score at second timing, median	2.00	5.00	.021*	0.798 (0.63-0.97)	0.007
Higher score in the second measure, n (%)	3 (21.4%)	10 (66.7%)	0.014†	-	-
Lower PLT count in the second measure, n (%)	9 (60%)	13 (81.3%)	0.252†	-	-
Higher CRP value in the second measure, n (%)	4 (28.6%)	12 (75%)	0.011†	-	-
Intubation between both measures, n (%)	0	7 (41.2%)	0.008‡	-	-
Respiratory score at suspicion, median	1.00	1.00	0.798*	-	-
Analytical score at suspicion, median	1.00	1.00	0.266*	-	-
Respiratory score at 2nd timing, median	1.00	2.00	0.296*	-	-
Analytical score at 2nd timing, median	1.00	3.00	0.001*	0.800 (0.62-0.98)	.006
